# Biomechanical Analysis and Design Method for Patient-Specific Reconstructive Implants for Large Bone Defects of the Distal Lateral Femur

**DOI:** 10.3390/bios12010004

**Published:** 2021-12-22

**Authors:** Po-Kuei Wu, Cheng-Wei Lee, Wei-Hsiang Sun, Chun-Li Lin

**Affiliations:** 1 Department of Orthopaedics, Therapeutical and Research Center of Musculoskeletal Tumor, Taipei Veterans General Hospital, Taipei 112, Taiwan; pkwu@vghtpe.gov.tw; 2 Orthopaedic Department School of Medicine, National Yang Ming Chiao Tung University, Taipei 112, Taiwan; 3 Department of Biomedical Engineering, National Yang Ming Chiao Tung University, Taipei 112, Taiwan; justinlee102185@ym.edu.tw (C.-W.L.); mickeysuen310.be10@nycu.edu.tw (W.-H.S.); 4 Medical Device Innovation & Translation Center, National Yang Ming Chiao Tung University, Taipei 112, Taiwan

**Keywords:** patient-specific implant, topology optimization, finite element analysis, bone cement, 3D printing

## Abstract

This study aims to develop a generalizable method for designing a patient-specific reconstructive scaffold implant for a large distal lateral femur defect using finite element (FE) analysis and topology optimization. A 3D solid-core implant for the distal femur defect was designed to withhold the femur load. Data from FE analysis of the solid implant were use for topology optimization to obtain a ‘bone scaffold implant’ with light-weight internal cavity and surface lattice features to allow for filling with bone material. The bone scaffold implant weighed 69.6% less than the original solid-core implant. The results of FE simulation show that the bone repaired with the bone scaffold implant had lower total displacement (12%), bone plate von Mises stress (34%), bone maximum first principal stress (33%), and bone maximum first principal strain (32%) than did bone repaired with bone cement. The trend in experimental strain with increasing load on the composite femur was greater with bone cement than with the bone scaffold implant. This study presents a generalizable method for designing a patient-specific reconstructive scaffold implant for the distal lateral femur defect that has sufficient strength and space for filling with allograft bone.

## 1. Introduction

Osteosarcoma of the distal femur is the most common primary sarcoma in adolescents [1]. At present, neoadjuvant chemotherapy before surgery, wide surgical resection of the tumor with limb salvage, plus adjuvant chemotherapy post-surgery are the standard clinical treatments. With such treatment, evidence suggests that the five-year survival rate is as high as 83% [2]. Wide resection involves removal of the tumor along with a 2–3-cm margin of normal tissue to prevent recurrence. This procedure results in considerable bone defects that require biological reconstruction. A liquid-nitrogen–inactivated autograft is commonly used for such reconstruction [3,4,5,6]. For larger tumors, bone defects remain after surgical reconstruction, making the overall support structure relatively fragile. In such cases, a prosthetic implant can be used to repair the defect. The ideal filler for reconstructing large bone defects should have a geometric shape matching that of the defect, provide structural strength, and allow for bone growth. While a variety of materials are available for bone defect implants, none is without drawbacks.

Poly (methyl methacrylate) (PMMA) bone cement is widely used for filling bone defects. The success of a cemented bone filler is strongly dependent on a reliable interface between the prosthesis and the cement as well as the mechanical bond between the bone and the cement. The inappropriate use of bone filling agents accounts for 14% of pathological fractures and 12–14% of bone non-union after surgery [7,8,9]. The interface between PMMA-based cement and the adjacent bone is typically weak, as PMMA bone cement is inherently bioinert: not bioresorbable, unable to be vascularized, and non-osteoconductive. Therefore, PMMA is not the optimal choice for filling bone defects, as it only provides structural support and does not facilitate the ultimate goal of bone ingrowth.

Bone grafting technologies using autograft tissue, allograft tissue, or artificially-produced materials have been proposed as alternative filling methods for reconstructing large bone defects. Such filling materials must be placed inside a scaffold that conforms to the shape of the defect and has sufficient strength to support the filler. In addition, the scaffold surface must have a lattice structure that allows for convenient placement of the filling materials inside the device during surgery. Specialized metal implants can be used for such reconstruction, providing a suitable scaffold for bone defect repair. Using image processing technology together with computer-aided design (CAD) and 3D additive manufacturing technology (also known as 3D printing), metal implants with complex tailor-made shapes can be successfully manufactured [10]. However, metal implants are heavy, and the higher elastic modulus of a metal implant can limit the load transferred to bone, causing stress shielding that can result in the cessation of bone growth [11]. Thus, the optimal implant structure must be lightweight and stabilized to allow for osteointegration and protected weight bearing [12,13,14,15].

Topology optimization is a mathematical method used by engineers to minimize the amount of material used and the strain energy on structures while maintaining their mechanical strength [16]. After finite element (FE) analysis to determine the main force-bearing area of a solid object, topology optimization is used to determine the volume to be retained and the specific areas that can be removed without decreasing its strength [17]. Using this method, customized lightweight implants have been designed to fill large defects in the supra-iliac spine [18], proximal tibia [19], and mandible [3]; simulation analysis of these implants showed that their biomechanical strength was no less than that of the solid implant. Such tailor-made implants are particularly needed for pediatric patients, as most standard implants are designed for adults; this lack of implant options can result in amputation rather than repair in this population [20]. While these previous studies report the successful engineering of implants optimized for a given patient, none describes a method generalizable to a variety of patients. In addition, no report describes the design of such an implant for tumor-related defects in the distal femur.

This study aims to develop a generalizable method for designing patient-specific reconstructive implants for large defects in the distal femur due to osteosarcoma. We used CAD to construct a solid 3D implant from computed tomography (CT) images, followed by FE analysis with topology optimization to design an implant with an internal cavity and lattices on the surface to decrease the implant weight. As the scaffold allow for the packing of autograft or allograft bone materials, this patient-specific implant is expected to have substantial mechanical strength while allowing for biological reconstruction via bone growth. The objective of this study was to compare the mechanical behavior for the distal lateral femur defect repaired using traditional PMMA cement with that of bone graft using patient-specific metal scaffold implant. In vitro biomechanical cyclic load tests were performed to compare the mechanical attributes of the metal scaffold implant to that of traditional bone cement filler.

## 2. Materials and Methods

### 2.1. Definition and Verification of Large Bone Defects in the Distal Femur

**Figure 1:**
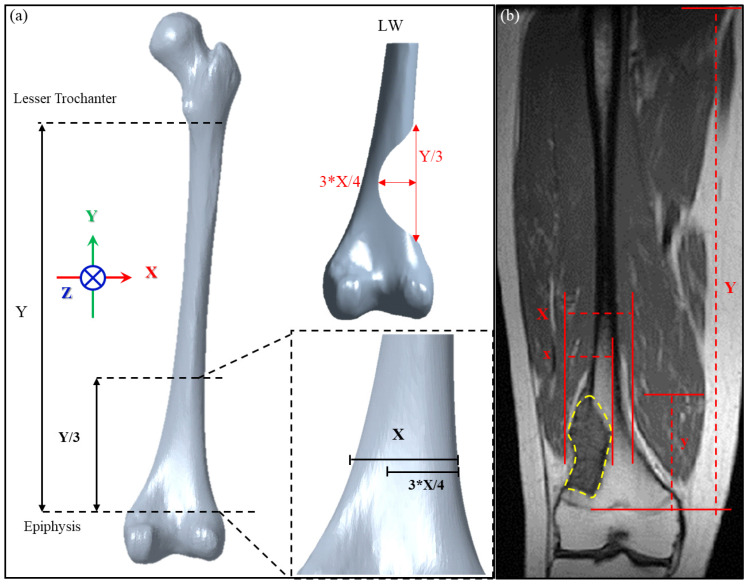
(**a**) Definition of the dimensions of the distal lateral femur defect according to femoral anatomical features; (**b**) application of the definitions to the MRI of a patient with a maximally large distal femur defect.

To establish criteria for designing patient-specific implant for the distal femur defect, we defined the defect dimension according to the anatomical features of the femur. For these criteria to be applicable, the femoral large-scale defect was assumed located above the epiphysis plate (this was the most common major defect from the image collected from patient with osteosarcoma at distal femur treating in our institute from 2015 to 2020). From the coronal perspective, the length of the femoral shaft from the lesser trochanter to the distal plate is defined as Y, and the defect is located at the ^Y^/_3_ position of the distal femur, which is simplified and assumed to be parabolic and symmetrical near the distal femur (Figure 1a). The maximum defect depth is located in the middle of the defect, and the femoral width at this position is defined as ‘X’. The defect depth is calculated as ¾ X. A schematic diagram of this large-scale defect, defined as LW defect in this study, is shown in Figure 1a.

The defect dimensions defined in this study were chosen as the most severe case possible for a distal lateral femur defect above the epiphyseal plate. Thus, the implant design will be broadly applicable to tumors at this location because defects larger than that investigated here will not be encountered in clinical practice. To verify the clinical applicability of our definition of a large-scale defect, we used image processing software (MICs 22.0, Materialise NV, Leuven, Belgium) to analyze the magnetic resonance image (MRI) of a tumor in a 32-year-old man to determine the tumor size range for which the design method is applicable. As shown in Figure 1b, the proportion of the tumor length y to the length Y between the lesser trochanter and distal epiphysis plate of the patient is 23.77%, which falls within the range of ^1^/_3_ Y (33.3% of Y). The proportion of tumor depth x compared to the femoral width X within this segment is 62.29%, which falls within the range of ¾ X (75% of X). Therefore, the definition of a large-scale defect established in this study includes this very extreme case and thus can be considered applicable to subsequent mechanical simulation analyses.

### 2.2. Implant Construction and Finite Element Analysis

**Figure 2:**
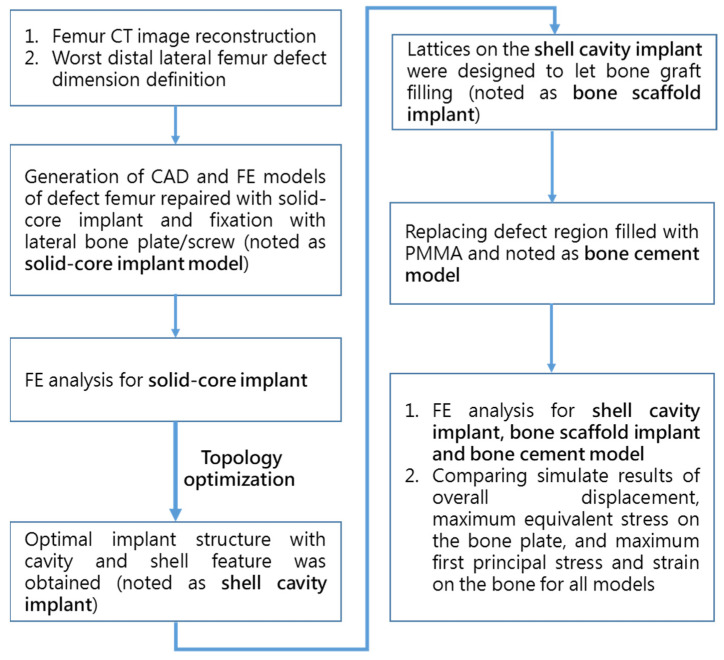
Flowchart of the sequence of FE analysis and in vitro biomechanical testing.

**Figure 3:**
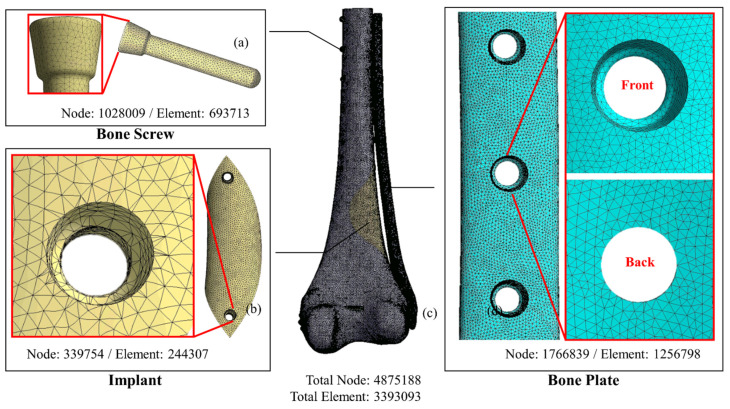
FE models of the system components. (**a**) bone screw; (**b**) solid implant; (**c**) femur; (**d**) bone plate.

A study flowchart of the sequence of FE analysis and in vitro biomechanical fatigue testing is shown in Figure 2. Before performing implant optimization analysis, it was necessary to conduct mechanical simulations according to the position of the solid-core implant in the distal femur. First, the CT image of the femur of a 12-year-old boy was imported, and a CAD model of the femur (including the corresponding cortical bone and internal cancellous bone) was constructed using Geomagic image processing software (Geomagic Studio, v12, Geomagic Inc., Morrisville, NC, USA). CAD software (PTC Creo, V6.0, PTC Inc., Needham, MA, USA) was used to cut out the distal femoral defect area conforming to the LW defect to generate a “solid-core implant” model of the corresponding defect. The distal and proximal ends of the solid-core implant were designed to lock and fix the implant to the femur with screws (diameter, 3.5 mm; length, 30 mm). The corresponding bone screw-plate fixation system (Tedray locking bone plate system, Quanwei Precision Co., Ltd., Taichung, Taiwan) was constructed on the lateral femur (Figure 3).

The CAD model of the femur/solid-core implant/implant screw/bone screw and bone plate system was imported into the computer-aided engineering analysis software (ANSYS, v19.0, ANSYS Inc., Canonsburg, PA, USA) for FE analysis. The properties of the materials used in the model, including cortical bone, cancellous bone, Ti6Al4V implant, and bone nail/bone plate system, were assumed to have linear elastic property with homogeneous and isotropy and corresponding Young’s modulus and Poisson’s ratio of different materials were assigned in the FE model (Table 1) [13]. The free mesh method using a tetrahedron element was adopted to generate the FE mesh model. The mesh size was as follows: femur, 1 mm; solid implant, 1 mm; bone nail, 0.5 mm; and bone plate, 0.5 mm (Figure 3).

**Figure 4:**
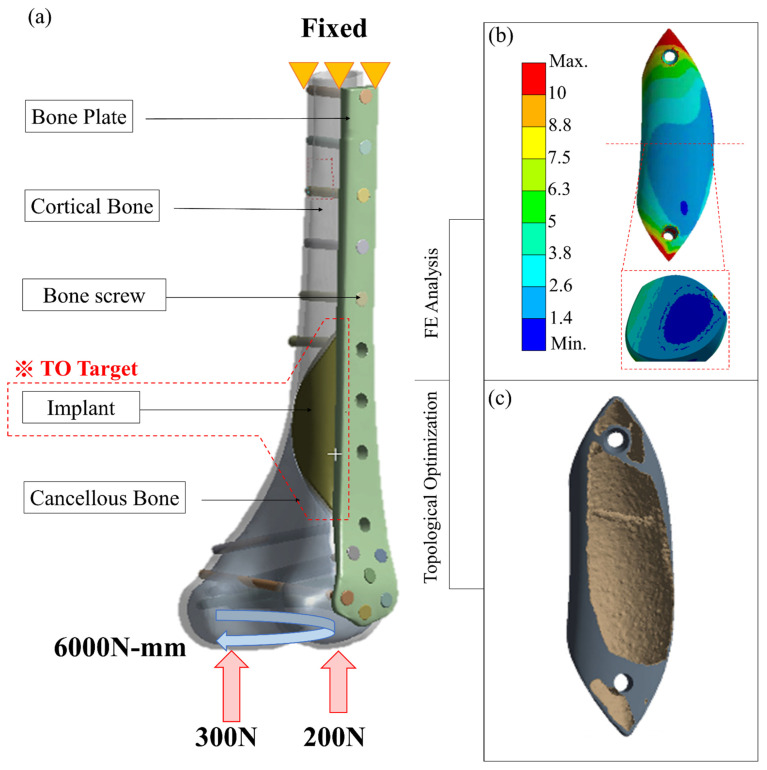
(**a**) Loading, boundary conditions, and material distribution of FE analysis for distal femur repaired with metal implant and external bone plate; (**b**,**c**) the result of topology optimization of the implant.

The contact surface was set as ‘No separation’ between the implant and the femur to simulate the situation in which a small dislocation is allowed but no separation is present. The bone screw/bone plate, bone screw/femur, implant screw/implant and implant screw/femur were all set as ‘Bond’ to simulate stress transfer continually. Patient weight (50 kg) was applied to the distal femur as the load condition (Figure 4a) to simulate the actual post-surgical stress on the femur while the patient is standing on one foot. The load was distributed as 60% (300 N) on the medial condyle surface and 40% (200 N) on the lateral condyle surface, with a rotating bending moment of 6000 N-mm. The proximal femur was fixed as the boundary condition in the analysis [4].

### 2.3. Topology Optimization and Biomechanical Analysis of the Implants

Using with data from the solid-core implant simulation, the topology optimization program in ANSYS was used to redesign the solid-core implant (Figure 4b) into the intermediate-model ‘shell cavity implant.’ Compliance minimization was used to ensure that the material retained after decreasing the weight was located in the main stress areas. The solid implant volume to be retained was set at 15%, with a 2-mm–thick shell surrounding an internal hollow cavity and the implant fixation screw.

**Figure 5:**
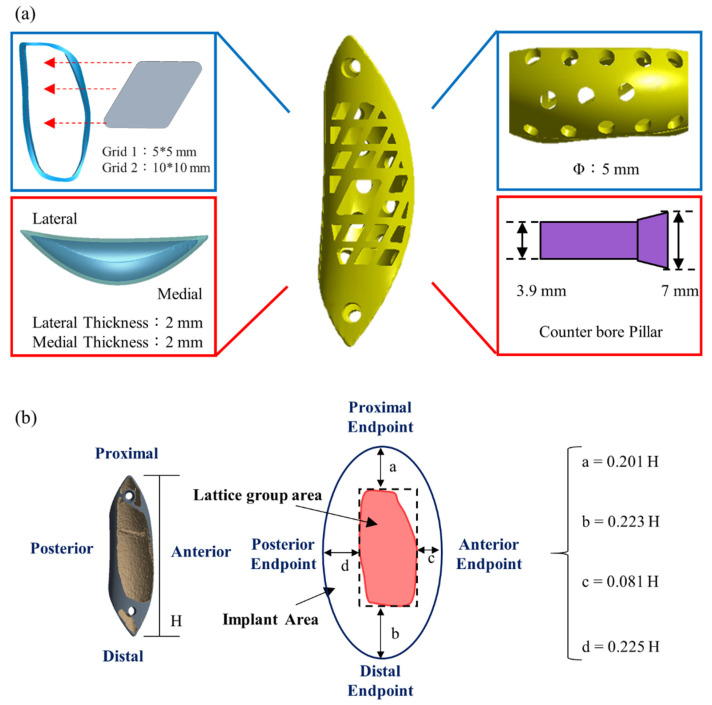
Features of the bone scaffold implant. (**a**) The outer surface was designed as a lattice structure to minimize stress and allow for filling with autologous or artificial bone; (**b**) The implant dimensions a, b, c, and d are reported relative to the implant length (Y/3) from the mesh area to the bone surface boundary.

To allow for the insertion of bone graft material into the implant, lattices were cut into the implant outer surface (Figure 5a). Generalizable design rules were set using dimensions a, b, c, and d of the implant (Figure 5b) to indicate distances from the lattice region to the bone surface boundary. These distances were defined relative to the implant length H as H = Y/3, where Y is the femur length (Figure 1). Most of the surface lattice was a 5-mm × 5-mm grid; a larger 10 mm × 10 mm grid was used for the larger bone, and the grid compartments were separated by 2-mm–wide stents. A number of 5-mm–diameter circular holes were made on the implant surface contacting the bone (inner side) to allow for bone growth. A counter hole was used where the fixing implant screw passes through the implant so that the screw lies flat on the implant surface when locked.

FE analysis was performed again for 4 femur-defect repair models: (1) filled with PMMA material (bone cement model); (2) Titanium alloy (solid-core implant); (3) Titanium shell cavity implant (shell-cavity implant) and (4) optimal titanium structure implant with surface lattice design (bone scaffold implant). All models were repaired using the bone screw-plate fixation system to compare overall displacement, maximum equivalent stress on the bone plate, and maximum first principal stress and strain on the bone under the same loading and boundary conditions.

### 2.4. Comparison of Biomechanical Parameters between Femurs Repaired with the Bone Scaffold Implant and Bone Cement

**Figure 6:**
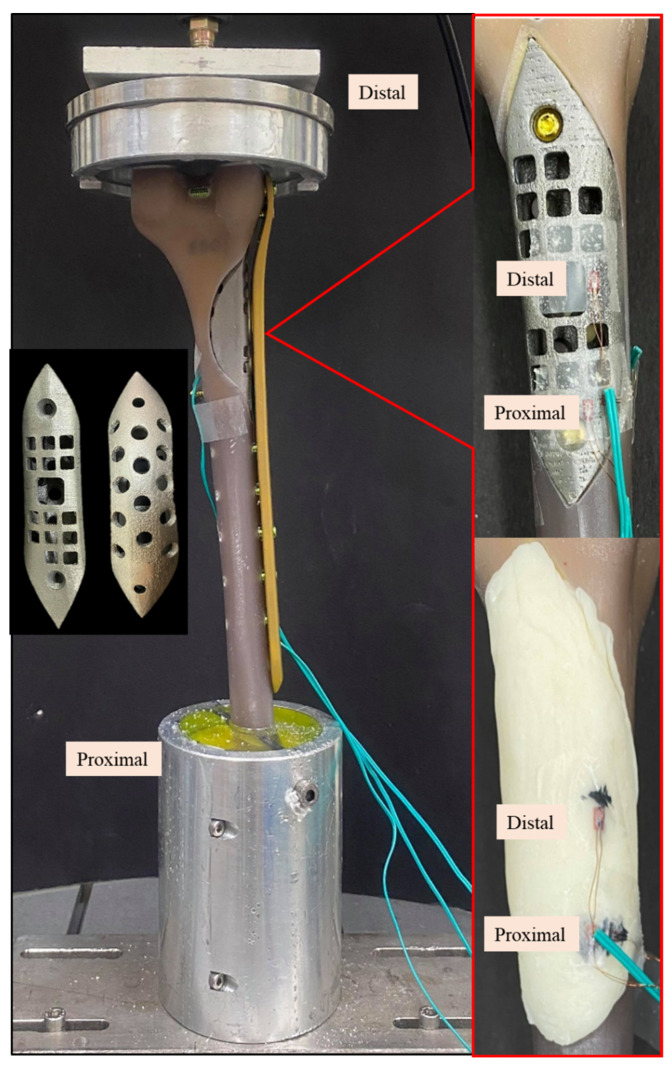
Photographic images of the Sawbone femur with a distal large defect repaired with a bone scaffold implant (**upper right**) and bone cement (**lower right**) under cyclic load test. Left middle image shows the top and back views of the bone scaffold implant manufactured by 3D printing.

In vitro biomechanical cyclic load tests were conducted as previously reported to compare the mechanical performance between femur defects repaired with a bone scaffold implant and bone cement [5,6]. The femur used for the experiment was made of composite synthetic bone (Femur, 4th Gen., Composite, 17 PCF Solid Foam Core, Small, Sawbones Inc., Vashon, WA, USA). Based on the defined LW defect size, a defect region was created in the synthetic femur using CNC (FMW-2513, FAIR Friend Enterprise Co., LTD., Taipei, Taiwan). A bone scaffold implant corresponding to the defect region was designed according to the methods described above and printed using metal additive manufacturing technology (metal 3D printer) (AM400, Renishaw plc, Woton-under-Edge, Gloucestershire, UK) (Figure 6).

Experiments were conducted in triplicate for each repair type, using 3 femurs repaired with a bone scaffold implant and 3 repaired with bone cement (Copolymer Bone Cement, Zimmer Inc., Warsaw, IN, USA). Strain was measured using two uniaxial strain gauges (Strain gauge N11, Showa Measuring Instruments, Tokyo, Japan) attached to the surface of the samples, as shown in Figure 6. After the implant was fixed to the composite femur, the bone screws (proximal: diameter, 5 mm; length, 26 mm; distal: diameter, 5 mm; length, 60 mm) and bone plate (length, 236 mm) were installed on the lateral surface (Tedrui locking bone plate system, Quanwei Precision Co., Ltd., Taichung, Taiwan). The proximal end was embedded and fixed onto the machine (Instron E3000, Instron, Canton, MA, USA) to facilitate the application of distal force, and the proximal and distal strain gauges on the bone scaffold implant/bone cement were connected to the mechanical strain measurement system (cDAQ 9178, NI Inc., Cary, NC, USA). The force was applied in nine stages (A–I) of increasing cyclic load (20,000 cycles/stage) (Table 2). The load was incrementally increased from 0.5–1 times the body weight to 0.5–5 times the body weight. At the end of one stage, the next stage began immediately, and each sample was subjected to 180,000 cycles in total. Finally, the changes in strains at the implant surface with the phased cyclic load was compared between the bone scaffold implant and bone cement. Measurements in response to load bearing were recorded every 5000 cycles; a total of 36 data points were taken for each sample.

## 3. Results

**Figure 7:**
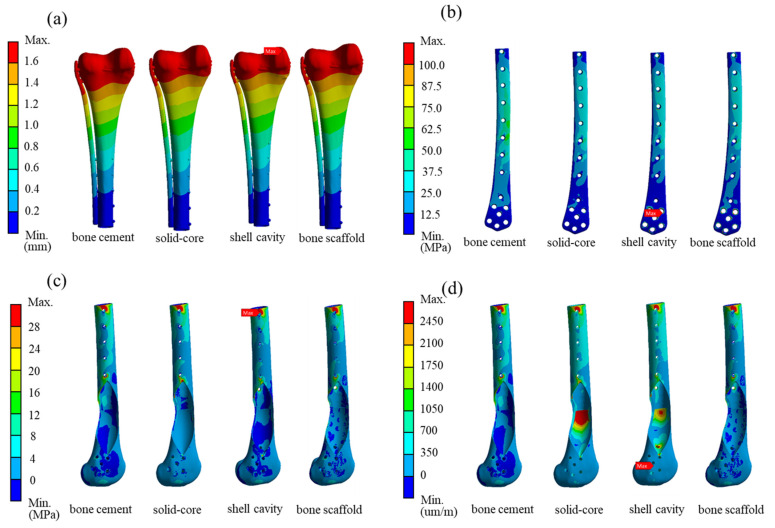
Simulation results for the distal femur defect repaired with solid-core, shell cavity, bone scaffold implants and bone cement. The magnitude and location of the stresses are indicated for (**a**) total displacement; (**b**) bone plate von Mises stress; (**c**) first principal stress on bone; and (**d**) first principal strain on bone.

The bone scaffold implant weighed 69.6% less than the solid-core implant. The simulation results for the bone scaffold implant, solid-core implant, shell cavity implant, and bone cement models are shown in Table 3. The total displacement, bone plate von Mises stress, maximum first principal bone stress, and maximum first principal bone strain differed by 10% or less between all implant models. However, greater variations in these parameters were observed between the bone scaffold implant and bone cement filler. The bone scaffold implant model was 12% lower in overall displacement, 34% lower in the maximum von Mises stress on the bone plate, 33% lower in the largest primary principal bone stress, and 32% lower in the largest primary principal strain compared to bone cement (Figure 7).

**Figure 8:**
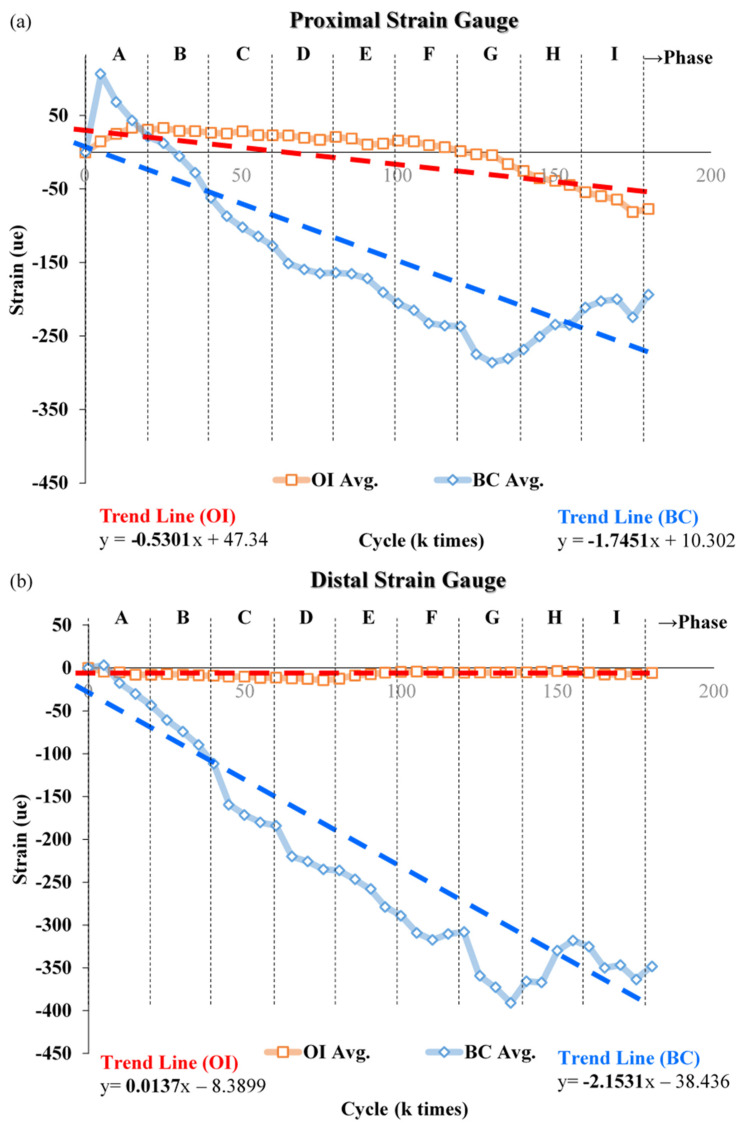
Change in strain with increasing load on femur repaired with the bone scaffold implant or bone cement. Across nine increases in load, the change in strain in the (**a**) proximal and (**b**) distal sites, strain values (negative) were greater in the bone cement than in the implant. Each data point is the average of 3 separate experiments.

The trends in strain with increasing load on repaired composite femurs in cyclic load tests are shown in Figure 8. The increase (negative) in proximal strain with increasing load stages was greater in the femur repaired with bone cement than with the bone scaffold implant, as indicated by the trend lines in Figure 8a. The slope of this line for bone cement was about three times that of the bone scaffold implant. The rate of change in distal strain in response to increasing load stage was clearly greater for the femur repaired with bone cement than with the bone scaffold implant, the trend line for which had a slope of approximately 0 (Figure 8b).

## 4. Discussion

This study established a generalizable method using FE analysis and topology optimization for designing patient-specific reconstructive implants for distal femur defects resulting from osteosarcoma. Using this method, we designed a titanium implant optimized for high mechanical performance, low volume, and low weight, with the capacity for osteointegration. The FE analysis of the 3D model created from a patient CT scan showed that compared to bone cement, the bone scaffold implant was lower in overall displacement and lower in the maximal von Mises stress on the bone plate and maximum first principal stress and strain on the bone. In vitro biomechanical tests showed that the increase in strain with increasing load stages was greater in femur repaired with bone cement than with the implant, and that the implant experiences minimal strain in response to load bearing. Thus, this design method can be used to produce optimized patient-specific reconstructive implants that impose less burden on the bone plate and bone.

Previous studies have combined CT imaging, CAD, FE analysis, and 3D printing to design implants for large defects in the supra-iliac spine [18], proximal tibia [19], and mandible [3], and for distal femur repair after traumatic injury [13]. However, these studies focused only on the implant design for a single case and did not generalize the design method or set rigorous design parameters to allow for applicability to other patients. To ensure that an implant product is safe and effective under the most severe structural conditions, the US Food and Drug Administration (FDA) requires that patient-specific reconstructive implants undergo pre-clinical testing, and the defect size range for which the product is suitable must be determined [3]. Thus, a method for determining this range for distal femur implants is needed to increase the availability of patient-specific reconstructive implants for distal femur defects. This study develops and assesses such a method using a case involving an exceptionally large deformity of the distal lateral femur to establish the outer contour condition with respect to size. The defect size was defined relative to the anatomical length and diameter of the patient’s own femur. Because this deformity was of the maximum possible size at this site, the fragility of the site in this specific case is expected to be the most extreme. An implant designed to suitably support the bone in this extreme case would therefore be suitable for defects of lesser size. Thus, individualized implants can be designed using the suggested general guidelines presented in this study.

Analysis of the bone scaffold implant designed in this study showed that topology optimization reduced the weight of the original solid implant by 69.6%, a primary aim for the optimization of metal implants, which are significantly heavier than bone. Implant weight reduction using topology optimization has been proven to reduce the elasticity modulus of the implant to avoid stress shielding [21]. FE analysis showed that the von Mises stress on the bone plate and the first principal stress/strain on the bone are greater for bone defects filled with bone cement than with the implant. This result can be explained with the Young’s modulus of the metal implant and the bone cement. With respect to material strength, a single force system consists of the force on each of the different materials in the system that bear the bending moment at the same time. Equation (1) was used to calculate the force endured by each material (*σ*, stress from bending moment; *E*, material 1; *E′*, elastic modulus of material 2; *I*, second axial moment of inertia the cross-sectional area; *y*, distance of the cross-sectional area neutral axis; and *A*, cross-sectional area).

(1) $$\sigma =-\frac{Ey}{I};\;\sigma dA=-\frac{Ey}{I}dA=-\frac{\left(nE^{\prime }\right)y}{I}dA=-\frac{E^{\prime }y}{I}\left(ndA\right)\;\text{where},\;n=\frac{E}{E^{\prime }}$$

First, scaling was required for one of the areas (volume), and the scaling ratio had to be in multiples of n between the Young’s modulus of the two materials to ensure a universal boundary condition for calculating the resultant force. Young’s modulus then was used to distribute each force so that the resultant force was directly proportional to the Young’s modulus.

**Figure 9:**
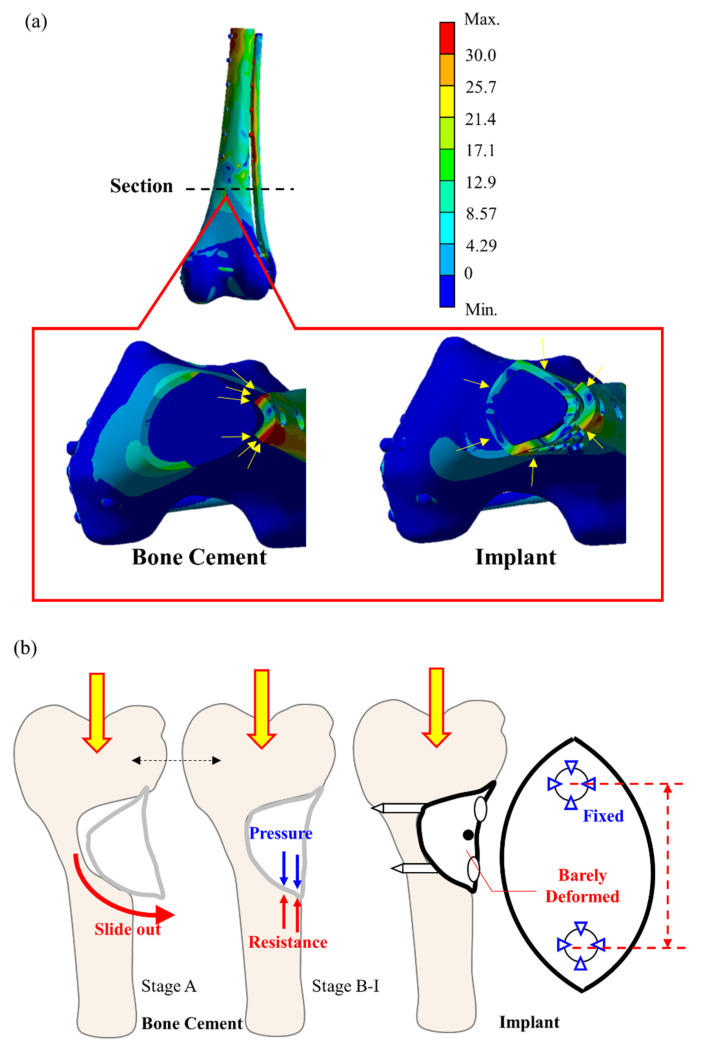
Stationary deformation analysis of bone cement and the bone scaffold implant. (**a**) the bone scaffold implant experienced minimal strain in response to load bearing as compared to bone cement; (**b**) the bone cement filler exerts a force that can cause distal prolapse, which is not seen in the bone scaffold implant.

The application of these calculations to determine the stress on the bone cement model and the implant is shown in Figure 9. In the bone cement model, the elastic modulus difference between the bone cement and Ti6Al4V is as high as 43 times, such that the stress is primarily borne by the external Ti6Al4V bone plate for the femur defect repaired with bone cement. Stress concentration did not occur in the bone cement region but was found at the bone plate (yellow arrow in the Figure 9a left). The stress was transmitted from the proximal bone screw to the external plate and back to the bone via the distal bone screw. This phenomenon induced high stress and strain in the bone surrounding the bone screw (Figure 7c,d). In contrast, for the bone scaffold implant, also made of Ti6Al4V, the bone plate stress was shared by the bone scaffold implant and transmitted through the implant in the defect area (Figure 9a). Therefore, no excessive stress concentration was present around the screw hole. This difference in load bearing between the implant and bone cement accounts for the marked difference in stress experienced by these materials. Thus, the bone scaffold implant is a more effective defect filler than is bone cement.

This study investigated the dynamic cyclic load experienced by the bone scaffold implant in vitro in response to increasing load as compared to that of bone cement. We observed that with lower load (at load stage A), the average strain on the proximal side femur increased in those repaired with either the bone scaffold implant or bone cement (Figure 8a). With lower loads, the lateral region of the defect deforms, bending inward due to tension. The bone cement filler exerts a tensile force at the lower cyclic load stage (A) that might cause cement block prolapse (Figure 9b). However, no apparent prolapse was observed in our experiment, indicating that the force applied during the cyclic test did not exceed the cement/saw bone binding force.

As the load force gradually increases (load stage B to I), the entire sample experiences a downward compression force. At this point, the tension in the lateral side is gradually replaced by the compression force, resulting in a gradual increase in compressive strain with subsequent increases in load. In response to increasing load, we observed that the rate of strain increase even in the proximal/distal femur was greater obviously in that repaired with bone cement than with the metal implant. We have speculated that this disparity results from differences in the Young’s modulus on each material and the way in which the filling material is fixed to the defect. In addition, the metal implant is secured to the femur with screws, which have a greater hardness and bear a greater force than bone cement. Thus, we observed a near-zero slope of effect in the distal strain site (Figure 8b).

This study has several limitations. Our FE analysis of the properties of each material assumed that each material was linear elastic, and the applied load consisted of a simple single weight. Under actual in vivo conditions, the femur receives greater feedback force from the muscles. In addition, the bone scaffold implant topology and reconstruction outcomes will be affected by the actual loading conditions. Nevertheless, the trends in the test results hold referential value, as the study maintained consistency in testing variables between the FE analysis and experimental cyclic load groups. This study provides a novel methodology that future studies could use to determine implant design guidelines for other bones and bone regions (e.g., lateral, proximal, and distal) to serve a wider variety of patients.

## 5. Conclusions

This study presents a generalizable method for designing patient-specific reconstructive implants for large bone defects of the distal femur due to osteosarcoma. The patient-specific bone scaffold implant designed in this study has a surface lattice design for filling with allograft bone, provides sufficient support, and is light-weight relative to solid-core implant. FE analysis and biomechanical cyclic load tests showed that the bone scaffold implant with outer surface lattice design experiences less displacement, bone-plate stress, bone stress/strain and experimental strain than does bone cement filling. Thus, this design method can provide an alternative option for developing patient-specific reconstructive implants for distal lateral femur defects.

## Figures and Tables

**Table 1:** Material properties used in FE analysis.

Material	Young’s Modulus (GPa)	Poisson’s Ratio	Reference
Cortical Bone	12.4	0.3	[13]
Cancellous Bone	0.104	0.3	[13]
Ti6Al4V	114	0.34	[13]
Bone Cement	2.65	0.455	[21]
Bone Graft	1	0.45	[22]

**Table 2:** The nine cyclic load stages applied to experimental femur-repair models.

Phase	Loading (100% BW)	Number of Cycles	Frequency (Hz)
A	0.5–1.0	20,000	2
B	0.5–1.5	20,000	2
C	0.5–2.0	20,000	2
D	0.5–2.5	20,000	2
E	0.5–3.0	20,000	2
F	0.5–3.5	20,000	2
G	0.5–4.0	20,000	2
H	0.5–4.5	20,000	2
I	0.5–5.0	20,000	2

**Table 3:** FE analysis of total displacement, bone plate von-Mises stress, bone maximum first principal stress and bone maximum first principal strain for all simulated models.

Group	Total Deformation (mm)	% Error Relative to Solid Implant	Bone Plate Von-Mises Stress (MPa)	% Error Relative to Solid Implant	Bone Maximum First Principal Stress (MPa)	% Error Relative to Solid Implant	Bone Maximum First Principal Strain (µm/m)	% Error Relative to Solid Implant
Solid-core Implant	2.039	-	310.2	-	38.15	-	2903	-
Shell cavity Implant	2.052	0.6%	335.2	8%	41.58	9%	3089	6%3
Bone scaffold implant	2.061	1%	313.0	1%	40.14	5%	2804	3%
Bone Cement	2.309	12%	420.1	34%	53.50	33%	3711	32%

## Data Availability

All data generated during the study can be found in this manuscript.
